# The ketogenic diet is not feasible as a therapy in a CD-1 nu/nu mouse model of renal cell carcinoma with features of Stauffer's syndrome

**DOI:** 10.18632/oncotarget.19306

**Published:** 2017-07-17

**Authors:** Silvia Vidali, Sepideh Aminzadeh-Gohari, René Günther Feichtinger, Renaud Vatrinet, Andreas Koller, Felix Locker, Tricia Rutherford, Maura O’Donnell, Andrea Stöger-Kleiber, Bridget Lambert, Thomas Klaus Felder, Wolfgang Sperl, Barbara Kofler

**Affiliations:** ^1^ Laura Bassi Centre of Expertise-THERAPEP, Research Program for Receptor Biochemistry and Tumor Metabolism, Department of Pediatrics, Paracelsus Medical University, Salzburg, Austria; ^2^ Department of Pharmacy and Biotechnology (FABIT), University of Bologna, Bologna, Italy; ^3^ Clinical Nutrition, Vitaflo International Ltd, Liverpool, UK; ^4^ Department of Laboratory Medicine, Paracelsus Medical University, Salzburg, Austria; ^5^ Department of Pediatrics, Paracelsus Medical University, Salzburg, Austria

**Keywords:** renal cell carcinoma, mitochondria, ketogenic diet, metabolism, Warburg effect

## Abstract

The ketogenic diet (KD), a high-fat low-carbohydrate diet, has shown some efficacy in the treatment of certain types of tumors such as brain tumors and neuroblastoma. These tumors are characterized by the Warburg effect. Because renal cell carcinoma (RCC) presents similar energetic features as neuroblastoma, KD might also be effective in the treatment of RCC. To test this, we established xenografts with RCC 786-O cells in CD-1 nu/nu mice and then randomized them to a control diet or to KDs with different triglyceride contents. Although the KDs tended to reduce tumor growth, mouse survival was dramatically reduced due to massive weight loss. A possible explanation comes from observations of human RCC patients, who often experience secondary non-metastatic hepatic dysfunction due to secretion of high levels of inflammatory cytokines by the RCCs. Measurement of the mRNA levels of tumor necrosis factor alpha (TNFα) and interleukin-6 revealed high expression in the RCC xenografts compared to the original 786-O cells. The expression of TNFα, interleukin-6 and C-reactive protein were all increased in the livers of tumor-bearing mice, and KD significantly boosted their expression. KDs did not cause weight loss or liver inflammation in healthy mice, suggesting that KDs are per se safe, but might be contraindicated in the treatment of RCC patients presenting with Stauffer's syndrome, because they potentially worsen the associated hepatic dysfunction.

## INTRODUCTION

Kidney cancer is one of the 10 most prevalent cancers in Western countries, accounting for approximately 2–3% of adult malignancies, and renal cell carcinoma (RCC) comprises approximately 90% of all kidney cancers [1, 2]. In patients with organ-confined disease, surgical resection is the standard therapy and has excellent outcomes [3]. Other current treatments, such as vascular endothelial growth factor (VEGF), platelet-derived growth factor (PDGF), and mammalian target of rapamycin (mTOR) antibodies and inhibitors, have been shown to increase progression-free survival, but the response is rather transient [4]. Moreover, RCC is often diagnosed at a late stage, when curative treatment is not possible. Indeed, metastatic RCC is highly resistant to treatment, with outcomes that are generally poor and a median survival after diagnosis of less than one year [1, 5].

Many tumor cells display a special metabolic signature characterized by high glucose uptake and aerobic glycolysis which, even in the presence of sufficient amounts of oxygen, prevents pyruvate from being metabolized by the respiration of mitochondria, namely oxidative phosphorylation (OXPHOS) [6–9]. This metabolic switch is known as the Warburg effect [10, 11]. In most cases this shift in metabolism is accompanied by a general down-regulation of OXPHOS activity [12–15], or it may involve deficiency of two or three of the OXPHOS complexes [16, 17], or a single defect of one of the OXPHOS subunits [18–20].

RCC also exhibits the Warburg effect. Indeed, in RCC, an increase in glycolytic proteins and depletion of several mitochondrial enzymes has been observed [12, 21]. Moreover, the more aggressive types of RCC are characterized by stabilization of hypoxia-inducible factor (HIF), even in normoxia, due to loss of function of the von Hippel-Lindau (VHL) gene. HIF also contributes to up-regulation of many glycolytic enzymes and suppression of mitochondrial glucose oxidation [4, 22].

The ketogenic diet (KD) is high in fat and low in carbohydrates and protein, and it mimics starvation or prolonged exercise without restricting energy intake. It is characterized by increased ketone body levels (e.g. acetoacetate and β-hydroxybutyrate) and reduced glucose levels in the blood. Because tumor cells highly depend on glucose for energy production, limiting the glucose supply by means of KD could have anti-tumor effects. Moreover, KD has been reported to foster immunity, reduce both inflammation and angiogenesis, and increase apoptosis [23–25]. Finally, KD has shown good potential in enhancing the sensitivity of cancer cells to chemotherapy and in protecting normal cells from radiotherapy. Thus, KD allows cancer treatment with lower doses of chemotherapeutic agents, which might also improve patient compliance [6, 26, 27].

KD was recently shown to be particularly effective in the treatment of brain tumors such as malignant glioma [25, 28], and was applied in several clinical studies as an adjuvant therapy for glioblastoma, astrocytoma, tumors of the gastrointestinal tract, and other advanced metastatic types of cancers [28–32]. In most cases the patients showed a stable disease or general clinical improvement, with increased progression-free survival. In a single case, there was tumor recurrence after KD suspension [24].

In preclinical studies, KD produced excellent results as an adjuvant therapy in the treatment of neuroblastoma in a murine xenograft model [6, 33]. Neuroblastoma and RCC share a similar metabolic signature, with reduced mitochondrial DNA content and a general reduction of OXPHOS activity [9, 12]. There is evidence that medium-chain triglycerides (MCTs) based KD's are as effective in the dietary management of intractable epilepsy as those based on long-chain triglycerides (LCTs) [34], and MCT is included in KD's as it is more rapidly metabolized and less likely to be stored in adipose tissue compared to LCTs [35, 36]. Based on these premises, we postulated that RCC patients might also benefit from KD therapy. Thus, to elucidate if KD can be used as a potential adjuvant in the treatment of RCC, we created xenografts of human RCC in immunodeficient mice and randomized the mice to a control diet group and to three KD groups with or without MCT enrichment.

## RESULTS

### Human RCC xenografts have similar respiratory features as human RCC

**Figure 1:**
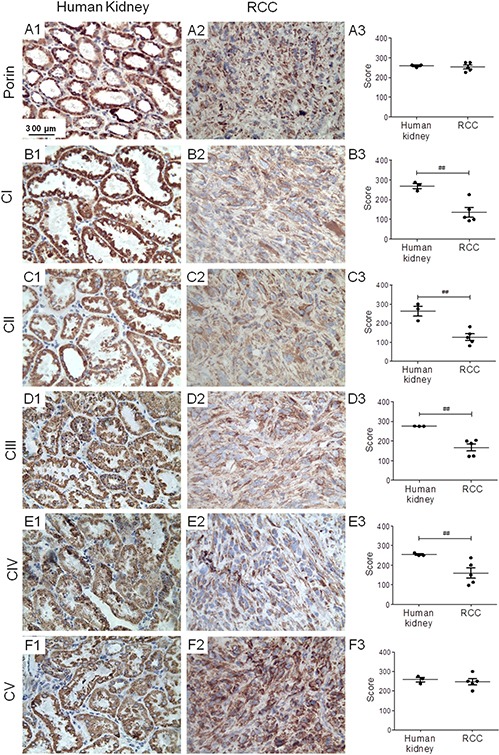
RCC shows reduced levels of OXPHOS complexes I–IV Immunohistochemical staining of porin and the OXPHOS complexes was performed (**A1–F1**) on normal human kidney, (**A2–F2**) and on RCC xenografts. (**A3–F3**) Graphs indicate score values of staining intensity. Data are given as mean ± SEM. Statistical analysis was performed by using student's *t* test (unpaired samples), ^##^*p* < 0.01; *n* = 3 (human kidney) and *n* = 5 (RCC). CI-CV, complexes I–V.

To ensure that human RCC xenografts of 786-O cells have similar respiratory features as human RCC, we carried out immunohistochemical (IHC) staining of the 5 OXPHOS complexes and porin (a marker of mitochondrial mass) on RCC 786-O xenograft tissue sections. The xenografts exhibited normal mitochondrial mass but significantly lower levels of OXPHOS complexes I–IV compared to normal kidney (Figure 1), consistent with previously published data on respiratory impairment in human RCC samples [12]. These results thus confirmed a general reduction of aerobic mitochondrial metabolism in the RCC 786-O xenograft model.

### Ketogenic diets reduce tumor growth but also overall survival of tumor bearing mice

**Figure 2:**
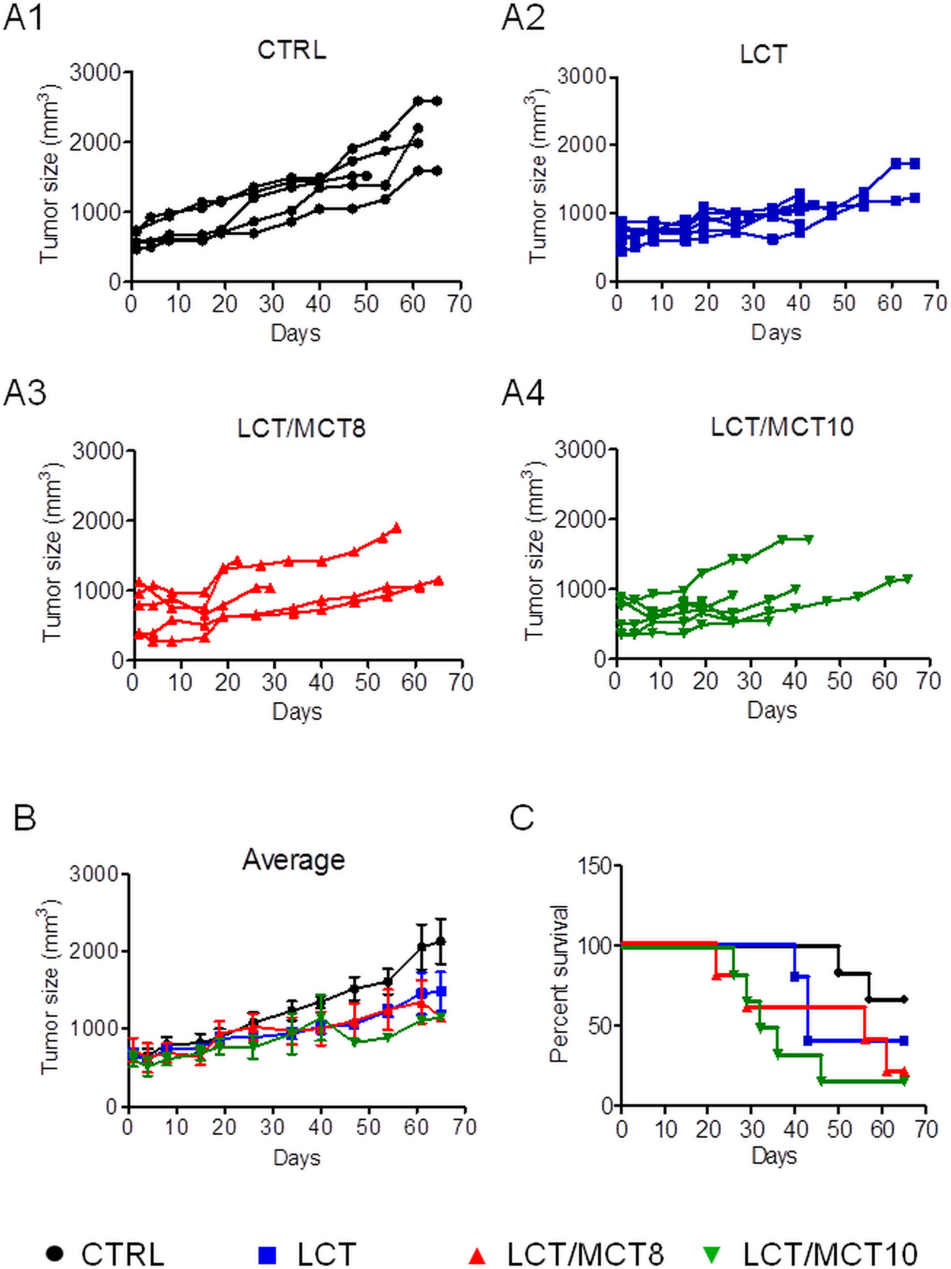
KDs generally caused a reduction in tumor growth, but also survival (**A1–A4**) Tumor growth in individual mice under different diets. (**B**) The graph shows the mean of the tumor mass during the treatment time. Data are given as mean ± SEM. Statistical analysis was performed by using two-way ANOVA (Dunnett's multiple comparison test), *n* = 5 for CTRL and LCT/MCT8 groups; *n* = 6 for LCT and LCT/MCT10 groups, at the start of the dietary intervention. (**C**) Kaplan-Meier survival curves for the RCC xenograft recipients treated with the different KDs. The statistical analysis for the survival curves was done with the Log-rank test (Mantel-Cox): CTRL vs. LCT, *p* = 0.2414; CTRL vs. LCT/MCT8, *p* = 0.1205; CTRL vs. LCT/MCT10, *p* = 0.0204.

All three KDs (LCT only; LCT/MCT8 – LCTs enriched with 25% 8-carbon MCTs; LCT/MCT10 – LCTs enriched with 25% 10-carbon MCTs) tended to slow down tumor growth compared to the control diet, although not significantly (Figure 2A1–2A4, 2B). Surprisingly, the KDs also tend to reduce the overall survival of the tumor bearing mice and this was significantly lower in the mice fed with LCT/MCT10. Mice that received the control diet (CTRL) showed 60% overall survival at the end of the experiment (65 days), those on the LCT diet showed 50% survival, whereas mice receiving the LCT/MCT8 and LCT/MCT10 diets showed the worst survival, with only 20% and 17% survivors, respectively (Figure 2C).

**Figure 3:**
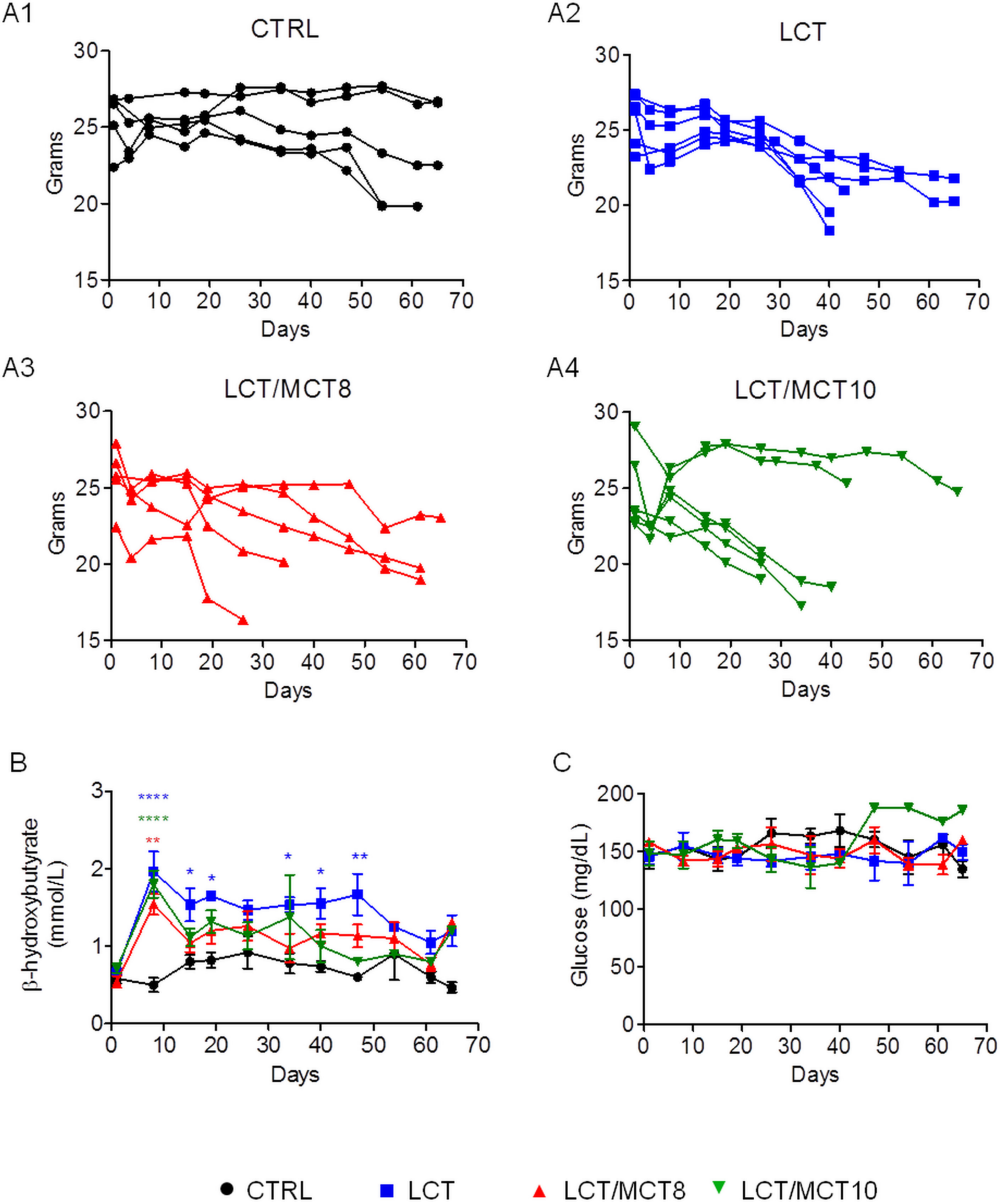
KDs boosted weight loss, increased blood ketone body levels at the beginning of therapy, and did not affect blood glucose levels (**A1–A4**) Body weight variations in individual RCC xenograft bearing mice during dietary intervention. (**B**) Average blood ketone bodies (mmol/L) and (**C**) average blood glucose (mg/dL) levels. Data are given as mean ± SEM. Statistical analysis was performed by using two-way ANOVA (not repeated measures, Bonferroni post-test), *p < 0.05, **p < 0.01, ***p < 0.001; *n* = 5 for CTRL and LCT/MCT8 groups; *n* = 6 for LCT and LCT/MCT10 groups, at the start of the dietary intervention.

Interestingly, mice with RCC xenografts frequently experienced massive and usually sudden weight loss, which was worse in all the KD groups compared to the CTRL group (Figure 3A1–3A4). Weight loss greater than 20% was the only reason for the early termination of treatment and euthanasia. Mice receiving either of the MCT-containing diets experienced pronounced weight loss. In the CTRL group mice maintained a fairly stable body weight until 50–60 days (Figure 3A1). In striking contrast, all tumor bearing mice receiving an LCT diet experienced weight loss after 26–30 days of treatment, and 67% of them lost more than 20% of their initial weight (Figure 3A2). In the LCT/MCT8 group, 40% experienced significant weight loss after 15–20 days (Figure 3A3) and in the LCT/MCT10 group, about 70% of the mice began to lose weight as early as 10 days after commencement of the treatment (Figure 3A4). Mice experiencing weight loss seemed a little lethargic (data not shown, subjective observation of the experimenter).

All three KDs provoked in the mice with RCC xenografts a pronounced and significant increase in the concentration of blood ketone bodies during the first week of treatment. The LCT group had a significantly higher blood ketosis until day 50, whereas that of the two LCT/MCT groups dropped to nearly normal levels after 15 days (Figure 3B). In contrast, average blood glucose levels remained quite stable throughout the 65 days of treatment (Figure 3C).

### Ketogenic diets are well tolerated by healthy CD-1 nu/nu mice

In light of the results obtained with RCC bearing mice, we tested whether the 8:1 KD per se induced altered food intake and weight loss, by feeding healthy mice for 40 days with CTRL, LCT, LCT/MCT8 and LCT/MCT10 diets. The food consumption in the KD groups was in average less, compared to the CTRL group (Supplementary Figure 1A). However, the average calorie intake was similar for the LCT and the CTRL groups and even significantly higher in the LCT/MCT8 and LCT/MCT10 groups (Supplementary Figure 1B). Importantly, the healthy mice did not experience weight loss in any of the dietary intervention groups; on the contrary they all stably gained weight. Only in the first 3–5 days from the beginning of the dietary intervention, mice in the LCT/MCT8 and LCT/MCT10 groups experienced a mild weight loss (Supplementary Figure 1C). Such a temporary weight loss is a normal consequence in the adaptation from one diet to another. In the first days the food intake in all diet groups was in average 1–1.5 grams/mouse/day and subsequently increased and stabilized to a daily intake per mouse of 3.4 ± 0.09 grams in the CTRL group, 1.9 ± 0.08 grams in the LCT group, 2.2 ± 0.15 grams in the LCT/MCT8 group and 2.4 ± 0.15 grams in the LCT/MCT10 group.

In contrast to the results obtained with the mice bearing the RCC xenografts, the healthy mice under the different KDs presented with a stable and sustained increase of blood ketone levels, compared to the CTRL fed mice (Supplementary Figure 1D), whereas glucose was not significantly affected by the LCT and the LCT/MCT8 diets, but mildly decreased in the LCT/MCT10 diet, compared to the CTRL group (Supplementary Figure 1E).

### KD does not affect common markers of liver dysfunction

Because patients with RCC often develop paraneoplastic syndrome with non-metastatic hepatic dysfunction [35, 36], liver dysfunction or inflammation could be the underlying cause of the weight loss seen in our xenograft model. Therefore, we analyzed biochemical markers of liver function in our experimental mice.

**Figure 4:**
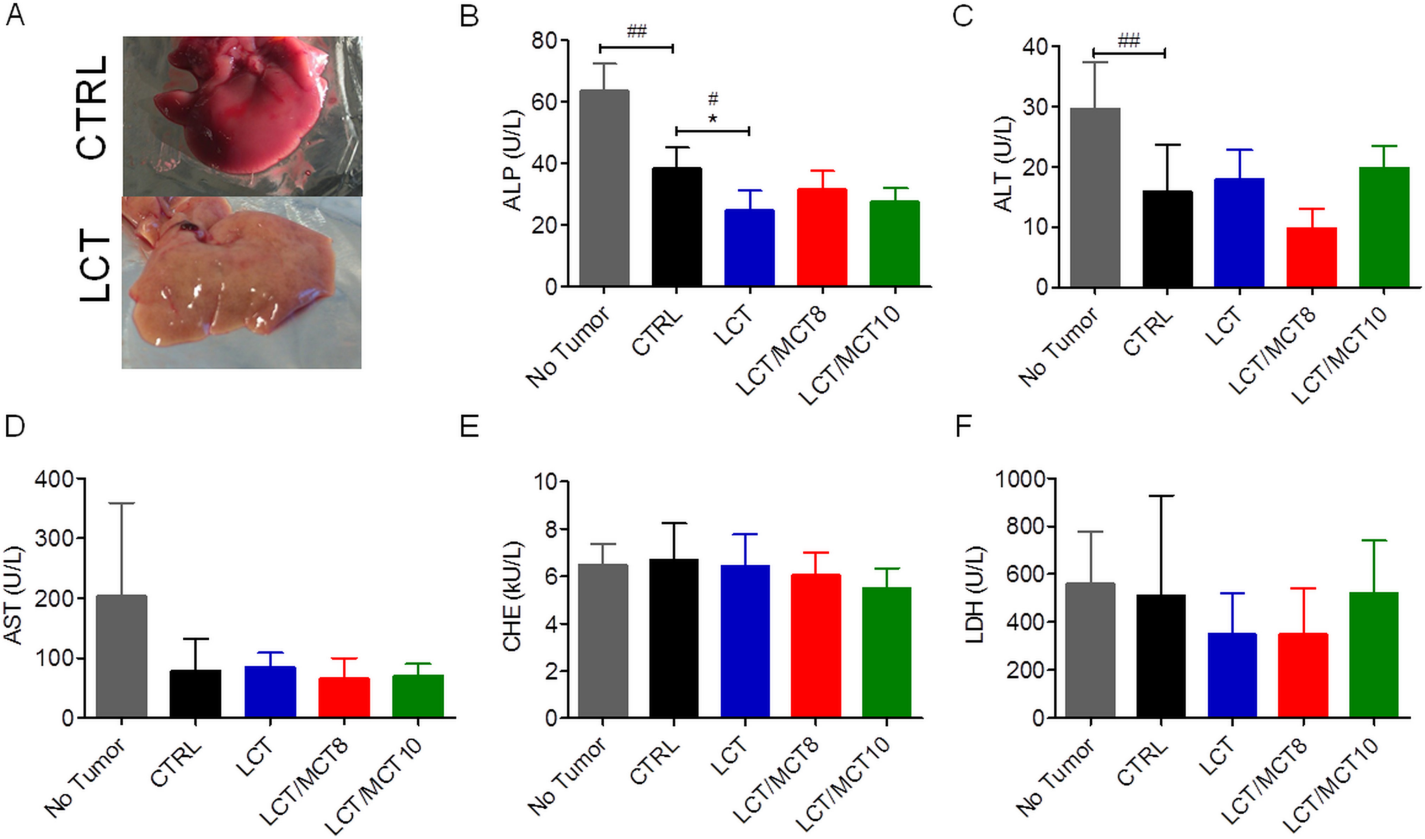
Liver enzymes were not increased in RCC xenograft recipients treated with a KD (**A**) Representative images of the liver from a tumor bearing mouse that received a CTRL diet and a tumor bearing mouse that received an LCT diet. Plasma levels of (**B**) alkaline phosphatase (ALP), (**C**) alanine aminotransferase (ALT), (**D**) aspartate aminotransferase (AST), (**E**) cholinesterase (CHE), (**F**) lactate dehydrogenase (LDH). Data are given as mean ± SEM, and statistical analysis was performed by using student's *t* test (unpaired samples) to compare CTRL vs. a certain KD, ^#^*p* < 0.05, ^##^*p* < 0.01, and one-way ANOVA (Kruskal-Wallis test) was performed to correct to multiple comparison, * *p* < 0.05; *n* = 5 for CTRL and LCT/MCT8 groups; *n* = 6 for LCT and LCT/MCT10 groups.

In some cases, the livers of mice bearing RCC in the KD groups exhibited a fatty appearance macroscopically (Figure 4A). However, we found no significant increase in any of the parameters routinely used as indicators of liver dysfunction, i.e., alkaline phosphatase (ALP), alanine aminotransferase (ALT), aspartate aminotransferase (AST), cholinesterase (CHE) and lactate dehydrogenase (LDH) (Figure 4B–4F). Moreover, although AST (Figure 4D), CHE (Figure 4E) and LDH (Figure 4F) were not significantly affected by the presence of the tumor or the type of dietary intervention, the levels of ALP (Figure 4B) and ALT (Figure 4C) were significantly reduced in xenograft-bearing mice compared to control mice without a tumor. Furthermore, the levels of ALP and ALT were not increased in the KD groups compared to the CTRL group; on the contrary, mice receiving the LCT diet had reduced ALP levels (Figure 4B).

To investigate whether the fatty appearance of the liver was due to the presence of fat droplets in the liver, we performed Oil Red O staining of hepatic sections (Supplementary Figure 2) of the xenograft bearing mice. The images framed in red show the liver parenchyma of mice that exhibited extreme weight loss. Only mouse 1 and mouse 2 in the LCT group showed a higher level of fat droplets, but the amount of red staining did not correlate with a reduction in survival.

### RCC is associated with increased mRNA levels of inflammatory cytokines in the liver

Because non-metastatic paraneoplastic syndrome of the liver is associated with inflammatory cytokines released by the tumor, e.g., interleukin-6 (IL-6) and tumor necrosis factor alpha (TNFα) [37–40], we examined the mRNA levels of these cytokines in both tumor tissue and the livers of xenograft and control mice. In addition, we measured the mRNA levels of C-reactive protein (CRP) in the liver, as CRP is a hepatic marker of the acute phase of inflammation, and its level depends on IL-6 secretion [41].

**Figure 5:**
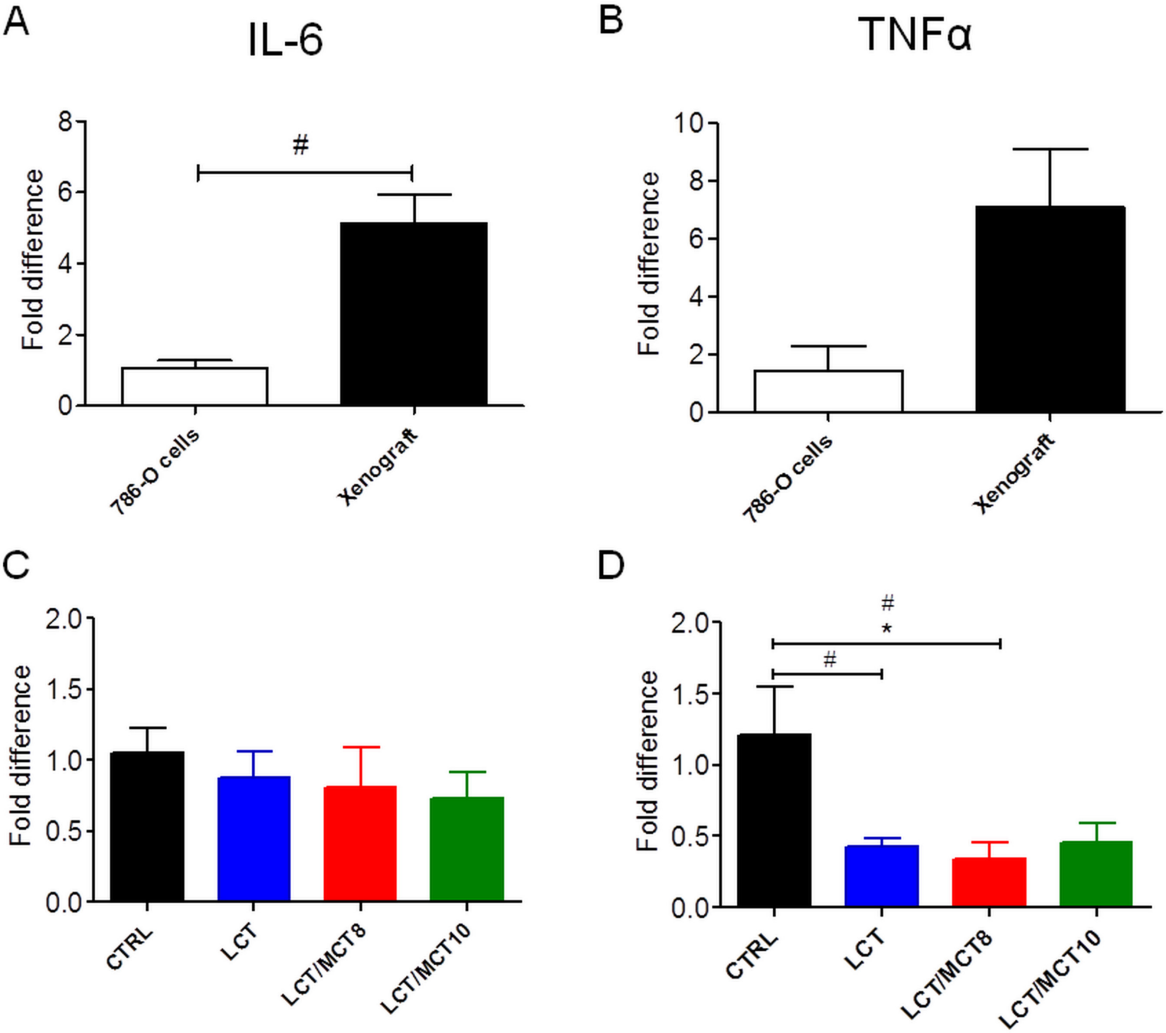
RCC xenografts expressed IL-6 and TNFα, but KDs did not increase their expression levels mRNA expression of (**A, C**) IL-6 and (**B, D**) TNFα in RCC xenografts. The xenografts in A and B are from mice fed with CTRL diet. Data are given as mean ± SEM. Statistical analysis was performed by using student's *t* test (unpaired samples) to compare CTRL vs. a certain KD, ^#^*p <* 0.05 and one-way ANOVA (Kruskal-Wallis test) was performed to correct to multiple comparison, *p < 0.05; *n* = 3 independent experiments for the 786-O cells; *n* = 5 for CTRL and LCT/MCT8 groups; *n* = 6 for LCT and LCT/MCT10 groups.

**Figure 6:**
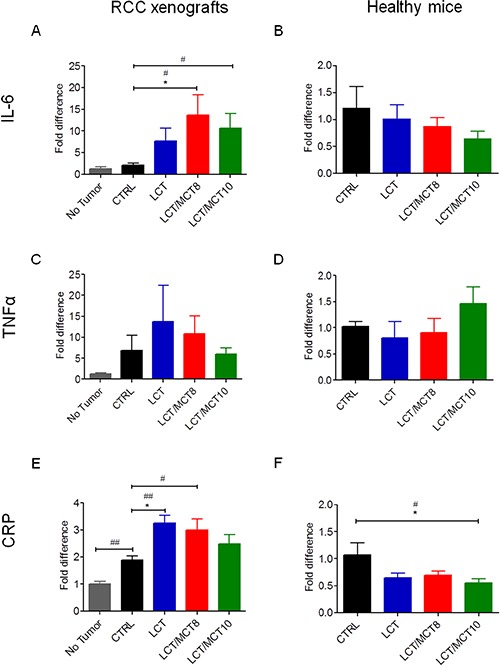
KDs tended to increase inflammatory cytokines in the livers of RCC xenograft recipients, but not in the livers of healthy mice mRNA levels of (**A, B**) IL-6, (**C, D**) TNFα and (**E, F**) CRP in liver tissue from (A, C, E) RCC bearing mice fed with CTRL or KDs compared to healthy mice fed with CTRL diet, and (B, D, F) from healthy mice fed with CTRL or KDs. Data are given as mean ± SEM. Statistical analysis was performed by using the student's *t* test (unpaired samples) to compare CTRL vs. a certain KD, ^#^*p <* 0.05, ^##^*p* < 0.01 and one-way ANOVA (Kruskal-Wallis test) was performed to correct to multiple comparison, *p < 0.05; (A, C, E) *n* = 4 for the no tumor group; *n* = 5 for CTRL and LCT/MCT8 groups; *n* = 6 for LCT and LCT/MCT10 groups; (B, D, F) *n* = 5 for CTRL and LCT groups; *n* = 6 for LCT/MCT8 and LCT/MCT10 groups.

The mRNA levels of TNFα and IL-6 were both high in the xenografts of mice under CTRL diet, compared to the original 786-O cells (Figure 5A, 5B). However, the xenografts in all three KD groups exhibited significantly decreased TNFα (but not IL-6) expression (Figure 5C, 5D). Interestingly, TNFα and CRP were both increased in the livers of mice bearing tumors compared to healthy control mice (Figure 6C, 6E). All three KD groups displayed significantly enhanced expression of IL-6 and/or CRP in the liver of the tumor bearing mice (Figure 6A, 6E). Also, a trend for elevated hepatic TNFα expression in the LCT and LCT/MCT8 groups was observed in the mice with tumor. These findings suggest that KD tends to promote inflammation and stress in the livers of RCC-xenograft bearing mice.

To demonstrate that the KDs per se were not causing liver damage, the same cytokines were also measured in the liver of the healthy mice fed with the CTRL diet or the KDs. Notably, the mRNA levels of IL-6, TNFα and CRP were not increased and the expression of CRP was even significantly decreased in the LCT/MCT10 fed healthy mice, compared to the CTRL fed healthy mice (Figure 6B, 6D, 6F).

## DISCUSSION

KD has been proven to be an effective adjuvant therapy in some types of solid tumors, including neuroblastoma, glioblastoma multiforme, and head and neck cancer [26, 28, 33, 42]. Furthermore, KD helps to protect normal cells from the toxicity of conventional therapies and it sensitizes tumor cells to the therapy, thus lowering the incidence of resistance [18, 19]. Besides affecting metabolism, KD has multiple effects on the regulation of inflammation and cell signaling, through its control of hormones and growth factors. Moreover, KD seems to prevent cachexia in mouse models of cancer [43, 44], making the KD an interesting option for the treatment of cancer patients, since advanced stage cancer patients can often experience cancer-induced cachexia [45, 46]. This makes KD a potent therapy for the treatment of many diseases; indeed, it is successfully used in the treatment of drug-resistant epilepsy in children [47, 48] and certain metabolic diseases such as pyruvate dehydrogenase deficiency and glucose transporter type 1 deficiency [49–51]. However, the precise mechanism(s) behind its efficacy is still being evaluated.

Our results suggest that RCC tumor bearing mice on a KD might experience reduced tumor growth; however, the data are not significant because of the low number of mice (< 3) that reached to the termination point of the experiment, due to the severe weight loss. Recently, another study reported that mice with xenografts generated with another RCC cell line (RXF393) also experienced severe weight loss, cachexia and muscle wasting [52], suggesting that the effect is tumor type-specific. Indeed, since the healthy mice fed with the KDs remained healthy throughout the dietary intervention, it is reasonable to conclude that the used KDs are not per se causing these symptoms.

Interestingly, one third of human patients affected by RCC experience a paraneoplastic syndrome. Symptoms can include anemia, polycythemia, hypercalcemia, hypertension, fever, cachexia and weight loss [53, 54]. One of the most common paraneoplastic events is a non-metastatic hepatic dysfunction called Stauffer's syndrome, which affects 10–15% of patients with RCC [36, 53]. Stauffer's syndrome is mainly associated with renal cancer and has rarely been reported in other types of tumors, such Hodgkin's lymphoma [55, 56] or soft tissue sarcoma [57, 58]. The hepatic dysfunction usually resolves after surgical removal of the tumor. The onset of liver dysfunction is mainly determined by cytokines released by the tumor, such as IL-6 and TNFα [37–39], and it generally provokes fever, weight loss and an unfavorable prognosis [59]. Interestingly, increased IL-6 and TNFα levels were associated *in vivo* and *in vitro* with reduced hepatic ketogenesis [60, 61], and this could explain the lower plasma ketone levels in the RCC bearing mice under KD, compared to healthy control mice.

IL-6 seems to have a broad range of effects on body metabolism and cancer. For example, in the liver it decreases gluconeogenesis, has anti-inflammatory effects, and shows anti-steatotic potential, but it also promotes liver carcinogenesis [62]. TNFα, together with other cytokines, seems to be an inhibitor of food intake, by regulating leptin release by the adipose tissue [63], or other hormones in the brain such α-melanocyte stimulating hormone [64], corticotropin releasing factor [65], and serotonin [66]. All these hormones stimulate anorexia and weight loss [67].

In our mouse model, we found that IL-6 was highly expressed in the tumor tissue and, although its expression in the tumors was not altered by the KDs, IL-6 mRNA levels were significantly increased in the livers of the tumor bearing mice that received the LCT/MCT8 and LCT/MCT10 diets compared to the CTRL group.

Similarly, TNFα expression was high in the tumor tissue, and it was also generally higher in the livers of tumor-bearing mice compared to healthy controls. Although the LCT and LCT/MCT8 diets were associated with reduced expression of TNFα in the xenografts, both diets tended to increase its expression in the liver of the mice with xenografts. However, when the KDs were administered to healthy mice, the expression of IL-6 and TNFα remained unaffected.

CRP is considered a negative marker for overall survival of cancer patients [68, 69]. Indeed, its expression is responsible for anemia in RCC patients. Importantly, CRP plasma levels rise in response to inflammation. It is an acute-phase hepatic protein, and the levels are increased by IL-6 secretion from macrophages and T cells. The final effect of CRP elevation is activation of the complement system [41].

In our RCC model, the livers of engrafted mice exhibited a significant increase in CRP mRNA levels compared to tumor-free mice. These levels were further increased in the KD-treated mice, indicating that the KDs potentiated this effect. On the contrary, the KDs did not increase the expression of CRP in the liver of healthy mice, and mice fed with LCT/MCT10 even showed a significant decrease of its expression, compared to the CTRL fed animals. This observation confirms that the KDs alone are not causing liver inflammation, and it confirms previous studies showing anti-inflammatory effects of KD [25, 70, 71], which might be mediated by Δ-hydroxybutyrate action [72].

Surprisingly, there was not a consistent increase in fat droplets in the livers of the xenograft recipients, nor were the plasma levels of biochemical markers of liver dysfunction increased. In fact, two of such markers (ALP and ALT) were decreased, granted a decrease in ALP can also be a sign of malnutrition, severe anemia and deficiency in magnesium and some vitamins [73]. However, normal liver function tests do not always mean the liver is normal. Patients with cirrhosis, liver fibrosis, or significant hepatic necroinflammation can sometimes present normal liver function tests. Only serum albumin, bilirubin and prothrombin time can provide more useful information on liver status [74, 75], but these tests require additional amounts of blood. However, this was not available due to the small volumes collectable from a single mouse. Thus, we could perform only the standard liver function tests.

Interestingly, a study performed on Eker rat models of tuberous sclerosis showed that long-term (8 months) administration of KD promoted renal tumor growth. Eker mice are characterized by mutations that provoke development of mostly non-neoplastic lesions in many organs [76]. This could have sensitized the kidney to cancer development, but still it suggests a higher susceptibility of the kidney to long-term administration of KD compared to other organs. Taken together, these findings suggest that KD per se does not show signs of toxicity, but that it might be contraindicated for the treatment of RCC, when associated with signs of Stauffer's syndrome. We can not exclude that using a lower fat:carbohydrate ratio than 8:1, might mitigate the effects on the liver and weight loss, this remains however to be tested.

Our study suggests that the feasibility of KD as an adjuvant cancer therapy strongly depends on the type of cancer and even its genetic alterations. For example, a recent study reported that KD increased tumor cell proliferation in BRAF positive melanoma but not in BRAF negative melanoma cells [77], confirming that KD has different effects depending on the type and genetics of a tumor. Anyway, one has also to be careful in translating results from animal models into the human practice, as in a human trial involving patients with different types of advanced cancer, a patient presenting with a BRAF positive melanoma was the one which benefited the most [78]. Of course it is impossible to draw conclusions from a single case, and in large scale studies the results could be different.

Thus, it is important to test the practicability of KD in specific tumor types in pre-clinical studies first, and then carefully translate them to human clinical trials.

## MATERIALS AND METHODS

### Cell culture

The most common and aggressive type of RCC exhibits loss of von Hippel-Lindau (VHL) factor and constitutive expression of hypoxic inducible factor (HIF); therefore, we chose 786-O (CRL-1932, ATCC, Germany) cells to establish RCC xenografts. Indeed, 786-O cells are characterized by a truncated form of HIF-1α and absence of VHL. In RCC, when an HIF-1α mutation is present, VEGF is controlled by constitutive expression of HIF-2α [79]. Cells were cultivated in high glucose RPMI-1640 medium (Sigma-Aldrich, Austria) supplemented with heat-inactivated fetal bovine serum (Gibco, Austria) and 100x penicillin/streptomycin amphotericin B solution (Lonza, Germany) diluted 1:100.

### Animal experiments

All *in vivo* experiments were performed in accordance with protocols approved for this study by the Salzburg Animal Care and Use Committee (No. 20901-TVG/87/7-2014). Animals were maintained under specific pathogen-free conditions and care conformed to the Austrian Act on Animal Experimentation. All experiments were performed on female CD-1 nu/nu mice (Charles River, Germany), the animals were group-housed and had unlimited access to food. Feeding of the healthy mice with the experimental diets started on 7 weeks old mice.

Food consumption was calculated by subtracting the grams of remaining food from the grams of added food, and then dividing by the number of mice present in the cage and the number of days; measurements were performed every 3–4 days.

Xenografts were established in 5- to 6-week-old mice by injection of 200 μl of a 1:1 suspension of 10^7^ cells in serum-free medium and matrigel into the right flank (BD Biosciences, Austria).

When tumor size reached 600–700 mm^3^, mice were randomized into different ad libitum dietary intervention groups (*n* = 5–6) and group-housed. Xenograft recipients were monitored twice a week for body weight using a digital scale, and tumor volume by using a caliper and calculating the volume according to the formula (width × height × length)/2. Blood glucose and ketone body (β-hydroxybutyrate) levels were monitored once a week using a specific enzyme-based kit (Precision Xceed, Abbott Laboratories, Austria). Measurements were performed after a two-hour fasting period.

For the xenografts, mice were euthanized 65 days after injection of tumor cells or when termination criteria were reached, such weight loss above 20% of the net body weight; none of the mice was scarified due to the tumor size, since none of the xenografts reached the termination size of 10% of the net mouse weight. One 5-mm thick slice from the central part of the tumor was formalin-fixed and paraffin-embedded for histological analysis, and the remaining cancer tissue was snap frozen in liquid nitrogen. Livers were also collected and snap frozen in liquid nitrogen. Finally, prior to being sacrificed, mice were injected with 10 μl/g of anesthetic mix (ketamine 20.5 mg/ml, xylazine 5.4 mg/ml, acepromazine 270 μg/ml in saline solution), and after checking for absence of reflexes from the paw, heart puncture was performed and blood was collected into tubes (BD Microtainer^®^ PST^TM^ LH tubes) (BD Biosciences, Austria). As suggested in the manufacture's protocol, tubes were inverted 10 times and centrifuged at 10000g for 90 seconds to separate plasma; plasma was then collected and snap frozen in liquid nitrogen.

The experiment on healthy animals was terminated after 40 days of treatment and the same protocol for the euthanasia and the sample collections used for the xenograft bearing mice was applied. All frozen samples were stored at −80°C until analysis.

### Dietary intervention

Mice were randomized into four dietary groups fed ad libitum: control diet (CTRL); long-chain fatty acid KD (LCT); 25% 8-carbon medium-chain fatty acids and 49.6% LCT KD (LCT/MCT8); and 25% 10-carbon medium-chain fatty acids and 49.6% LCT KD (LCT/MCT10) (Sniff Spezialdiäten GmbH, Germany). Moreover, diets were fortified with vitamins and minerals (Table 1).

**Table 1:** Composition and energy supply of the different diets

	CTRL	LCT	LCT/MCT8	LCT/MCT10
Crude Protein %	16.1	8.1	8.1	8.1
LCT %^a^	7.1	74.6	49.6	49.6
MCT8 %^b^	0	0	25	0
MCT10 %^b^	0	0	0	25
Sugar %	6	1	1	1
Starch %	51.2	0	0	0
Crude fiber %	10	9.9	9.9	9.9
Crude ash %	4.5	4.4	4.4	4.4
**Energy Kcal**	**3609**	**7098**	**7098**	**7098**
	per Kg	per Kg	per Kg	per Kg
Vitamin A (IU/IE)	15	15	15	15
Vitamin D_3_ (IU/IE)	1.5	1.5	1.5	1.5
Vitamin E (mg)	150	150	150	150
Vitamin K_3_ (mg)	20	20	20	20
Vitamin C (mg)	30	30	30	30
Copper (mg)	11	11	11	11

CTRL, control; LCT, long-chain triglyceride; MCT8, 8-carbon medium-chain triglyceride; MCT10, 10-carbon medium-chain triglyceride.

^a^LCT composition: butter fat 11.7% and pork lard 88.3%

^b^MCT10 and MCT8 composition: pure oil

### Murine plasma screening for liver dysfunction

The plasma collected from the mice after sacrification was analyzed for common liver function markers (alkaline phosphatase, alanine aminotransferase, aspartate aminotransferase, cholinesterase and lactate dehydrogenase) by standard laboratory methods on a COBAS8000 instrument (Roche Diagnostics, Germany) [80].

### (Immuno-)histochemical staining

Immunohistological stainings were performed on 4-μm deparaffinized sections. Human control kidney tissue was obtained from the Department of Pathology (Paracelsus Medical University, Salzburg). For the immunohistochemistry of porin and OXPHOS complexes I–V the following antibodies were used: mouse monoclonal anti-complex I subunit NDUFS4 (1:1000; Abcam, UK), mouse monoclonal anti-complex II subunit SDHA (1:3000; Abcam, UK), mouse monoclonal anti-complex III subunit core 2 (1:1000; Abcam, UK), mouse monoclonal anti-complex IV subunit I (1:1000; Abcam, UK), mouse monoclonal anti-complex V subunit-α (1:2000; Abcam, UK) and mouse monoclonal anti-voltage-dependent anion channel (VDAC1)/porin (1:2000; Abcam, UK). All antibodies were diluted in Dako antibody diluent with background reducing components (Dako, Denmark). The immunohistological stainings were performed as previously described [81].

A scoring system was used to quantify differences in expression levels between tumor and control human kidney tissue, as follows: the staining intensity (0: no staining; 1: weak staining; 2: moderate staining; 3: strong staining) was multiplied by the mean percentage of immunopositive cells. The score for each section was calculated as the mean of 4 high-power fields.

Oil Red O staining was performed on 6-μm thick cryosections. A stock solution was prepared by dissolving 3.75 g Oil Red O powder (Sigma-Aldrich, Austria) in 1000 ml of 2-propanol (Merck-Millipore, Germany). The working solution was prepared by adding 40 ml of distilled water into 60 ml of stock solution, and filtering through a Whatman^®^ filter (Sigma-Aldrich, Austria). Cryosections were taken from the −80°C storage, air dried for 10 min and then fixed for 20 min in 4% formaldehyde solution (Merck-Millipore, Germany). After fixation, the sections were washed twice for 5 min with distilled water and air dried for another 10 min. Slides were then dipped into 50% ethanol (Merck-Millipore, Germany), followed by a 20-min incubation in Oil Red O working solution and more dips into 50% ethanol. Afterwards, the slides were rinsed in distilled water and incubated for 3 min in Hematoxylin solution (Merck-Millipore, Germany). Finally, the slides were washed for 5 min under running tap water and mounted using Aquatex^®^ (Merck-Millipore, Germany).

### Quantitative real-time PCR

RNA of tumor and liver tissue was isolated using TRI-Reagent (Molecular Research Center, USA) following the manufacturer's protocol. Genomic DNA contamination was eliminated with the Turbo DNA-free Kit (Thermo Scientific, Austria) according to the manufacturer's instructions. Maxima Reverse Transcriptase (Thermo Scientific, Austria) and random hexamer primers were used for cDNA synthesis as recommended in the manufacturer's protocol. For gene quantification, iQ SYBR green supermix (Bio-Rad, USA) was used. Primers were synthesized by Microsynth (Switzerland). Target sites of the forward and reverse primers were separated by at least one intron (Human: IL-6: fwd 5′- AGATGT AGCCGCCCCACACAG -3′, rev 5′- CCAGTGCCTCTTT GCTGCTTTCA -3′; TNFα: fwd 5′- CCTGCTGCACTTT GGAGTGA -3′, rev 5′- CTTGTCACTCGGGGTTCGAG -3′; Ribosomal protein L 27 (RPL27): fwd 5′-GCTGGAA TTGACCGCTACC-3′, rev 5′-TCTCTGAAGACATCC TTATTGACG-3′; murine: IL-6: fwd 5′- CCGGAGAGGAG ACTTCACAGAGG -3′, rev 5′- TCTGCAAGTGCATC ATCGTTGT -3′; TNFα: fwd 5′- GGTCCCCAAAGGGAT GAGAA -3′, rev 5′- CTCAGCCACTCCAGCTGCTC -3′; CRP: fwd 5′- TCCCAGCAGCATCCATAGCCA -3′, rev 5′- TGGCTTCTTTGACTCTGCTTCCA -3′; RPL4: fwd 5′- GTATGGCACTTGGCGGAAGG -3′, rev 5′- TG CTCGGAGGGCTCTTTGG -3′). The amplification reaction was performed for 45 cycles (97°C for 15 seconds, 63°C human or 64°C mouse for 30 seconds and 72°C for 10 seconds) in duplicates. The relative expression of the genes was determined by the difference between the threshold cycle (Ct) of the gene of interest and the Ct of the housekeeping gene RPL27 for human and RPL4 for murine transcripts.

### Statistical analysis

Statistical analyses were performed using Prism 6 (GraphPad Software, USA). Results are given as mean ± SEM. Student's *t* test (unpaired samples) was applied to compare two groups, one-way ANOVA (Kruskal-Wallis test) or two-way ANOVA (not repeated measures, Bonferroni post-test), to compare more than one group and correct for multiple comparison.

## SUPPLEMENTARY MATERIALS FIGURES



## Abbreviations

ALP: alkaline phosphatase
ALT: alanine transferase
AST: aspartate aminotransferase
CHE: cholinesterase
CRP: C-reactive protein
CTRL: control
HIF: hypoxia inducible factor
IL-6: interleukin-6
KD: ketogenic diet
LCT: long-chain triglyceride
LDH: lactate dehydrogenase
MCT8: 8-carbon medium-chain triglyceride
MCT10: 10-carbon medium-chain triglyceride
RCC: renal cell carcinoma
RPL: ribosomal protein L
TNFα: tumor necrosis factor alpha
VHL: von Hippel-Lindau.
