# Supplementary material for: The ketogenic diet is not feasible as a therapy in a CD-1 nu/nu mouse model of renal cell carcinoma with features of Stauffer's syndrome
Source: Oncotarget. 2017 Jul 17;8(34):57201–15. doi: 10.18632/oncotarget.19306 (PMC5593636; doi:10.18632/oncotarget.19306)
Supplement: Supplementary file 1 [file oncotarget-08-57201-s001.pdf]

The ketogenic diet is not feasible as a therapy in a CD-1 nu/nu mouse model of renal cell carcinoma with features of Stauffer's syndrome

SUPPLEMENTARY MATERIALS

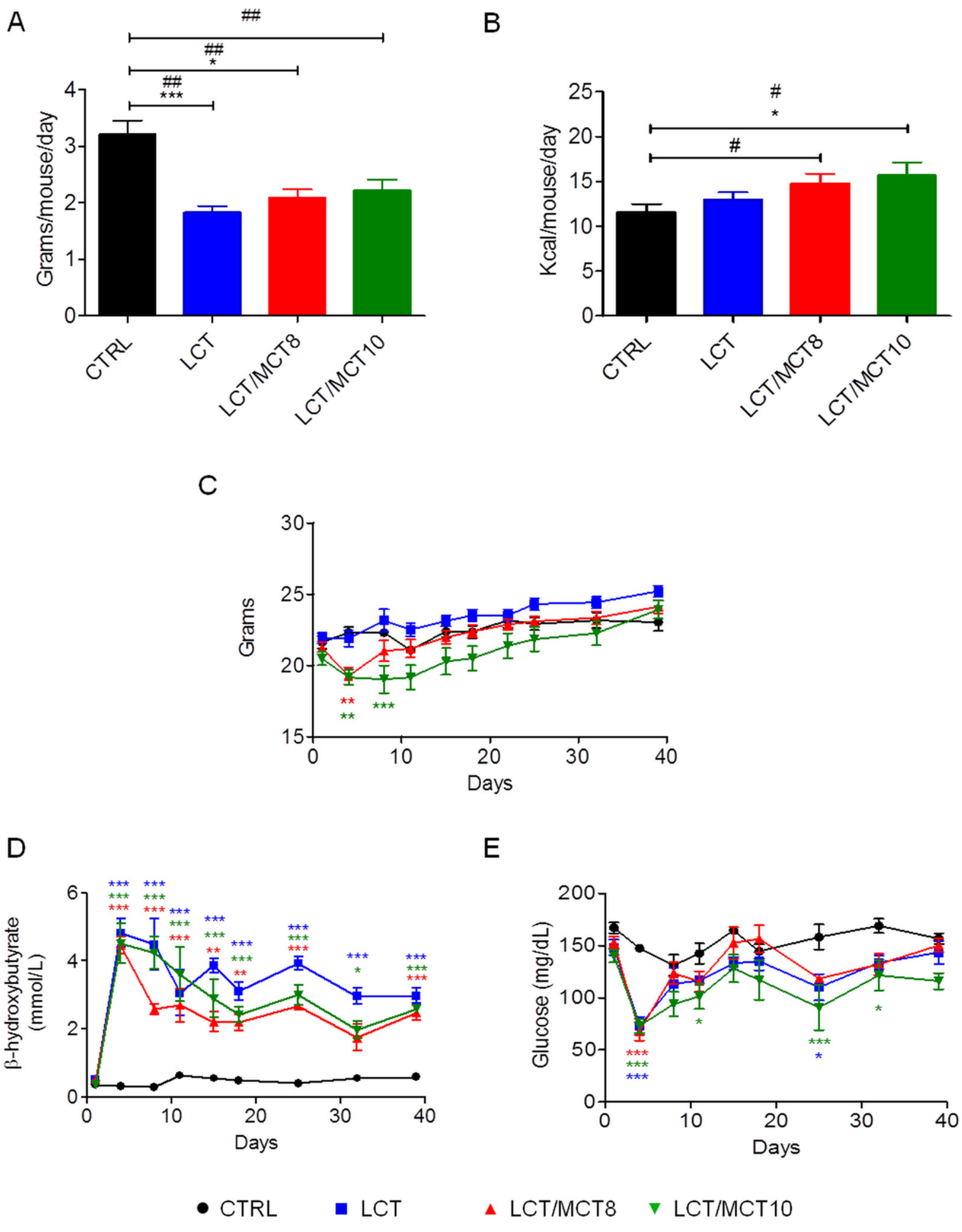

Supplementary Figure 1: KD did not induce weight loss in healthy animals and stably increased blood ketone body levels. (**A**) Average grams of food consumed per mouse per day. (**B**) Average daily calorie (Kcal) consumption of mice in different diet groups. Data are given as mean ± SEM. Statistical analysis was performed by using the student's *t* test (unpaired samples) to compare two groups of dietary intervention, $^{\#}p < 0.05$, $^{\#\#}p < 0.01$ and one-way ANOVA (Kruskal-Wallis test) was performed to correct to multiple comparison, $^{*}p < 0.05$, $^{***}p < 0.001$; $n = 10$ measurements per diet group. (**C**) Average body weight variations in mice during dietary intervention. (**D**) Average blood ketone bodies (mmol/L) and (**E**) average blood glucose (mg/dL) levels. Data are given as mean ± SEM. Statistical analysis was performed by using two-way ANOVA (Dunnett's multiple comparison test), $^{*}p < 0.05$, $^{**}p < 0.01$, $^{***}p < 0.001$; $n = 6$.

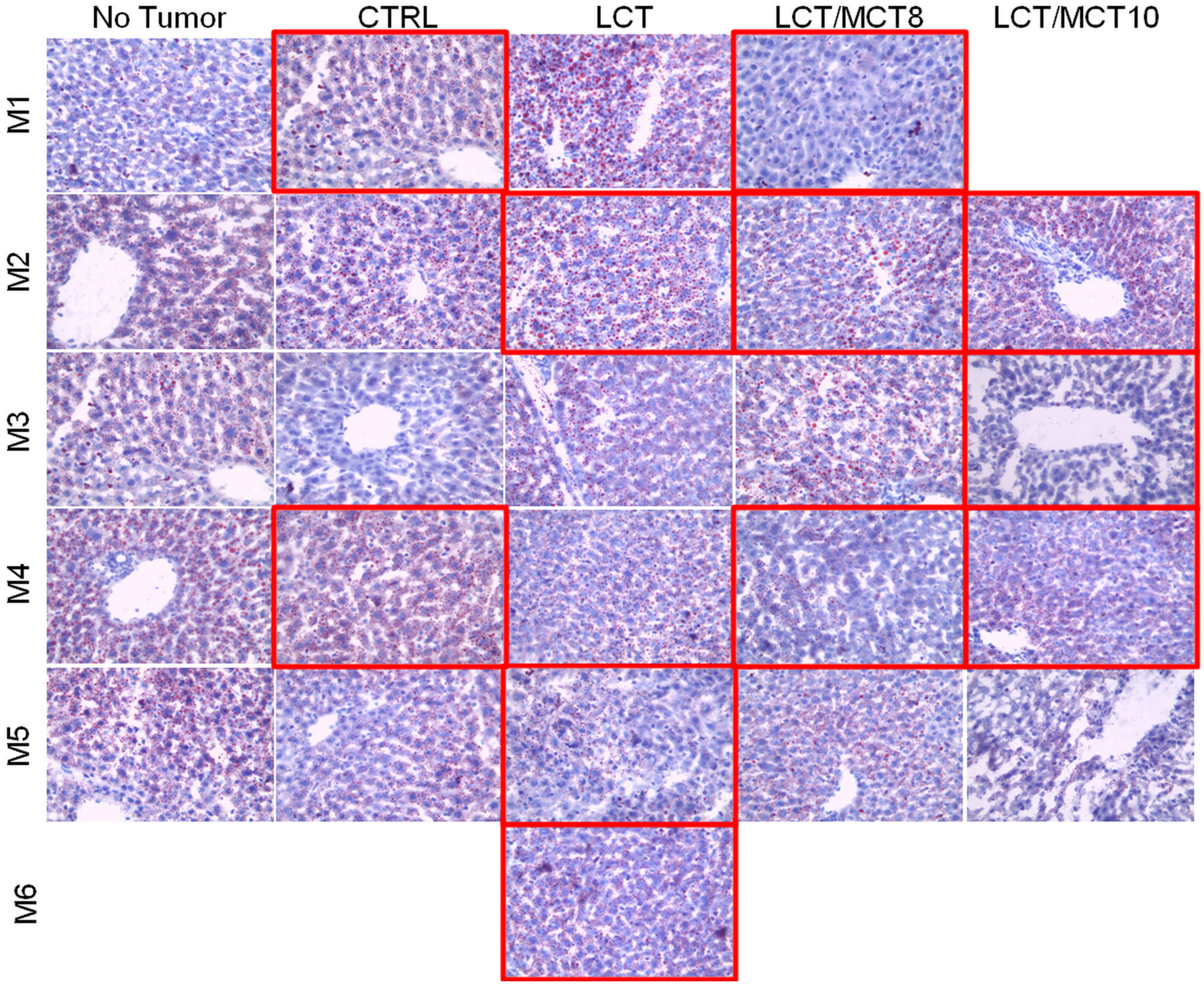

Supplementary Figure 2: Fat droplets were not increased in RCC xenograft recipients treated with a KD. Oil Red O staining for the detection of fat droplets on cryosections of mouse liver. The red frames indicate mice that experienced severe weight loss.
